# Antimicrobial Activity of Some Essential Oils—Present Status and Future Perspectives

**DOI:** 10.3390/medicines4030058

**Published:** 2017-08-08

**Authors:** Sonam Chouhan, Kanika Sharma, Sanjay Guleria

**Affiliations:** Natural Product Laboratory, Division of Biochemistry, Faculty of Basic Sciences, Sher-e-Kashmir University of Agricultural Sciences and Technology, Main Campus Chatha, Jammu, Jammu and Kashmir 180 009, India; sonam.chouhan.3007@gmail.com (S.C.); sharmakanika.bch@gmail.com (K.S.)

**Keywords:** essential oils, antimicrobial, synergy, nano-encapsulation

## Abstract

Extensive documentation on the antimicrobial properties of essential oils and their constituents has been carried out by several workers. Although the mechanism of action of a few essential oil components has been elucidated in many pioneering works in the past, detailed knowledge of most of the compounds and their mechanism of action is still lacking. This knowledge is particularly important for the determination of the effect of essential oils on different microorganisms, how they work in combination with other antimicrobial compounds, and their interaction with food matrix components. Also, recent studies have demonstrated that nanoparticles (NPs) functionalized with essential oils have significant antimicrobial potential against multidrug- resistant pathogens due to an increase in chemical stability and solubility, decreased rapid evaporation and minimized degradation of active essential oil components. The application of encapsulated essential oils also supports their controlled and sustained release, which enhances their bioavailability and efficacy against multidrug-resistant pathogens. In the recent years, due to increasingly negative consumer perceptions of synthetic preservatives, interest in essential oils and their application in food preservation has been amplified. Moreover, the development of resistance to different antimicrobial agents by bacteria, fungi, viruses, parasites, etc. is a great challenge to the medical field for treating the infections caused by them, and hence, there is a pressing need to look for new and novel antimicrobials. To overcome these problems, nano-encapsulation of essential oils and exploiting the synergies between essential oils, constituents of essential oils, and antibiotics along with essential oils have been recommended as an answer to this problem. However, less is known about the interactions that lead to additive, synergistic, or antagonistic effects. A contributing role of this knowledge could be the design of new and more potent antimicrobial blends, and understanding of the interplay between the components of crude essential oils. This review is written with the purpose of giving an overview of current knowledge about the antimicrobial properties of essential oils and their mechanisms of action, components of essential oils, nano-encapsulated essential oils, and synergistic combinations of essential oils so as to find research areas that can facilitate applications of essential oils to overcome the problem of multidrug-resistant micro-organisms.

## 1. Introduction

In the light of developments made in the scientific field, the medicinal properties of plants have received a great interest because of their low toxicity, pharmacological activities and economic viability [1]. Such studies have focused on the benefits of plant-extracted phytochemicals and their effect on human health. Additives obtained naturally from plants can be compounds, groups of compounds, or essential oils. In the recent times, there has been an increase in the food industry’s interest in natural compounds either for direct addition or for use in synergy with other compounds. It has been reported that direct addition of aromatic plant essential oils and extracts to foodstuffs exert an antioxidant or antimicrobial effect [2]. Among compounds of natural origin, biological activities have been shown by essential oils from aromatic and medicinal plants and have received particular attention because of their radical-scavenging properties [3]. Several pathologies such as cancer, deterioration of the organoleptic and hygienic quality of food, and neurodegenerative diseases have been attributed to free radicals [4]. Massive use of antibiotics has resulted in the emergence of resistance against them, which is another problem affecting public health [5]. *Pseudomonas aeruginosa*, *Staphylococcus aureus*, *Salmonella* spp, coagulase-negative *Staphylococcus*, *Shigella*, *Enterococcus sp.* and *Escherichia coli* are amongst some of the main bacteria with multidrug resistance and are included in the category of community and hospital acquired pathogens. This has resulted in the strong demand of new antibiotics by consumers against pathogens [6] and an interest has been developed by the scientific community for using herbal medicines with antimicrobial properties. Plants and other natural sources can provide a huge range of complex and structurally diverse compounds. Plant extracts and essential oils possess antifungal, antibacterial, and antiviral properties and have been screened on a global scale as potential sources of novel antimicrobial compounds, agents promoting food preservation, and alternatives to treat infectious diseases [7,8]. Essential oils have been reported to possess significant antiseptic, antibacterial, antiviral, antioxidant, anti-parasitic, antifungal, and insecticidal activities [9,10,11]. Therefore, essential oils can serve as a powerful tool to reduce the bacterial resistance [12]. Aromatic oily liquids called essential oils (also called volatile oils) are obtained from plant materials (leaves, buds, fruits, flowers, herbs, twigs, bark, wood, roots and seeds). A steam or hydro-distillation method was first developed in the middle ages by Arabs, and is the most common method for commercially obtaining essential oils. Having a density generally lower than that of water, essential oils are volatile, liquid, limpid, lipid soluble, rarely colored, and soluble in organic solvents. Being natural mixtures of very complex nature, essential oils may consist of about 20–60 components at quite different concentrations. Essential oils are characterized by two or three major components being present at fairly high concentrations (20–70%) in comparison to other components that are present in trace amounts. The amount of the different components of essentials oils varies amongst different plant parts and different plant species as they are chemically derived from terpenes and their oxygenated derivatives i.e., terpenoids that are aromatic and aliphatic acid esters and phenolic compounds. An important characteristic of essential oils and their components is hydrophobicity, which enables them to partition with the lipids present in the cell membrane of bacteria and mitochondria, rendering them more permeable by disturbing the cell structures. This eventually results in the death of bacterial cell due to leakage of critical molecules and ions from the bacterial cell to a great extent. Some compounds modulate drug resistance by targeting efflux mechanisms in several species of Gram-negative bacteria [13]. 

An important role of essential oils in nature is protection of plants by acting as antifungal, antibacterial, antiviral, and insecticidal agents and also protection against herbivores by reducing appetite of herbivores for plants with such properties. Health and Human Services Public Health Services have recognized essential oils as safe substances and some essential oils contain compounds that can be used as antibacterial additives [12]. Efficacy of essential oils (EOs) has been reported in several studies against pathogens and food contaminants [14], suggesting their applications in the food industry [10,11]. 

## 2. Antimicrobial Activity of Essential Oils

In recent years there has been a growing interest in researching and developing new antimicrobial agents from various sources to combat microbial resistance. Therefore, greater attention has been paid to the screening of antimicrobial activity and its evaluation methods. 

Several bioassays such as well diffusion, disk-diffusion, and broth or agar dilution are well known and commonly used methods [15]. The lowest concentration of antimicrobial agent that completely inhibits growth of the organism in micro-dilution wells or tubes as detected by the unaided eye is called minimum inhibitory concentration (MIC) [16]. The most appropriate bioassays for the determination of MIC value are these dilution methods, as these bioassays offer the possibility of estimating the concentration of the tested antimicrobial agent in the agar (agar dilution) or broth medium (macro dilution or micro-dilution) (Table 1). The most common estimation of bactericidal activity is the determination of minimum bactericidal concentration (MBC) which is defined as the concentration killing 99.9% or more of the initial inoculums [17]. 

Biological activity of essential oils from five Lamiaceae species, namely, *Mentha piperita*, *Lavandula angustifolia*, *Mentha pulegium*, *Salvia lavandulifolia and Satureja montana* was determined by Nikolíc et al. for their antimicrobial, cytotoxic properties and chemical composition. *Pseudomonas aeruginosa*, *Streptococcus pyogenes*, *Streptococcus mutans*, *Streptococcus sanguis*, *Streptococcus salivarius*, *Enterecoccus feacalis* and *Lactobacillus acidophilus* were the seven bacterial species, representing clinical specimens, along with fifty-eight clinical oral *Candida* spp. isolates with three reference strains used in the study. *Satureja montana* essential oil proved to be the most potent, and also significant antimicrobial activity was exhibited by all essential oils against all tested microorganisms [18]. In another study, the antibacterial activity of the essential oil from dried leaves of oregano (*Origanum vulgare*) that were fully formed and leaves and flowers of lavender (*Lavandula officinalis*) was reported. The lowest values of minimum inhibitory concentration were yielded by oregano essential oil against *E. coli* with an MIC value of 1600–1800 ppm, whereas the MIC value of lavender essential oil was 2000 ppm. On the other hand, MIC value of oregano essential oil was 800–900 ppm and the MIC value of lavender essential oil was 1000–1200 ppm against *S. aureus*. The higher content of phenolic compounds was reported to be the cause for this inhibition [19]. Aumeeruddy-Elalfi et al. evaluated the antimicrobial properties of essential oils against eighteen microorganisms (bacterial and fungal isolates) that were isolated from seven exotic and two endemic medicinal plants of Mauritius. Using the micro broth dilution assay, significant antibacterial activities were recorded with low minimal inhibitory concentration for eight essential oils except for *Salvia officinalis*, where the recorded activity was comparable with the activity of antibiotics [20]. It has been reported that the antibacterial activity of fruit of *Eucalyptus globulus* showed an inhibition effect against the pathogenic strains *Pseudomonas aerugenosa*, *Staphylococcus aureus*, *Bacillus subtilis*, *Escherichia coli* and *Listeria innocua*, with an MIC value of 3–4 mg mL^−1^. The MBC value of bactericidal effect varied between 3.6 and 9.0 mg mL^−1^, which demonstrated that all the tested bacteria were sensitive to the essential oil of *Eucalyptus globulus* fruits [25].

Likewise, it was reported that the secondary essential oil (SEO) of *Mentha citrata* showed antibacterial activity against all eight tested bacterial strains of Gram-positive bacteria, namely, *Staphylococcus aureus* (MTCC 96), *Staphylococcus epidermidis* (MTCC 435) and *Streptococcus mutans* (MTCC 890), as well as Gram-negative bacteria, namely, *Pseudomonas aerugenosa* (MTCC 741), *Klebsiella pneumoniae* (MTCC 109), *Escherichia coli* (DH5α), *Escherichia coli* (MTCC 723) and *Salmonella typhimurium* (MTCC 98), with an MIC value of 50–1000 µg/mL, while primary essential oils (PEOs) were active against seven strains with an MIC value of 250–1000 µg/mL [26]. Furthermore, a recent study assessed antimicrobial activity of six commonly used Brazilian condiments, viz., *Ocimum basilicum* L. (basil), *Rosmarinus officinalis* L. (rosemary), *Origanum majorana* L. (marjoram), *Mentha piperita* L. var. Piperita (peppermint), *Thymus vulgaris* L. (thyme) and *Pimpinella anisum* L. (anise) against a *Clostridium perfringens* strain. The MIC value for thyme essential oil was 1.25 mg mL^−1^ and 5.0 mg mL^−1^ for both marjoram and basil essential oil. Similarly, the three condiments, namely peppermint, rosemary and anise, showed MIC values of 10 mg mL^−1^. With the exception of anise oil which was only bacteriostatic, bactericidal activity was shown by all the oils at their respective MICs [21]. Tomáš et al. evaluated the antibacterial activity of the essential oil of *Epilobium parviflorum* Schreb against five microorganisms (*Pseudomonas aeruginosa*, *Staphylococcus aureus*, *Enterococcus faecalis*, *Escherichia coli*, and *Candida albicans*) using a microdilution method. Inhibition in the growth of all tested bacteria was observed. 

An increase in bacterial resistance to antibiotics and the lack of new antibiotics introduced into the market resulted in a need to find alternative strategies so as to cope with infections resulting from drug-resistant bacteria [22]. Development of alternatives for antibiotics and the discovery or development of adjuvants are amongst the potential strategies proposed [27]. In order to increase or restore antimicrobial efficacy against multi-drug-resistant bacteria, some efforts have been made. Addition of essential oils to antibiotics can induce a reduction in the antimicrobial MIC and the maximum effect has been observed with aminoglycosides, such as amikacin [28]. It has been shown in assays that geraniol demonstrates good activity in modulating drug resistance of various Gram-negative bacterial species (*Enterobacter aerogenes*, *E. coli*, *P. aeruginosa*) by targeting efflux pumps and could restore susceptibility to drugs in strains that over-express efflux pumps. This modification of drug resistance by EOs is more evident for drugs such as chloramphenicol, β-lactams and fluoroquinolones [29]. *S. aureus* is a common Gram-positive bacterium that can cause pathogenic conditions including food-borne diseases and infections ranging from minor localized skin disturbances to life-threatening deep tissue and systemic illness. Different components of EOs obtained from *Alpinia pahangensis*, *Origanum vulgare*, *Origanum dictamnus*, *Mentha piperita*, *Lavandula hybrida*, *Zataria multiflora* and *Hofmeisteria schaffneri* have been tested against *S. aureus*, and all were found to possess potential inhibitory activity [30,31]. Furthermore, combination of essential oil with an antimicrobial agent produces a synergistic effect against multidrug-resistant *S. aureus*, and in many cases, a substantial decrease in the MIC has been observed [32].

## 3. Mechanism of Action

The most appropriate method for determining the bactericidal effect as well as a strong tool for obtaining information about the dynamic interaction between the anti-microbial agent and the microbial strain is the time-kill test. Also a time-dependent or a concentration-dependent antimicrobial effect is revealed by the time-kill test.

Li et al. reported that the kinetic curves (antibacterial) of *Litsea cubeba* oil at 0.0625% (*v*/*v*) was able to prolong the lag phase growth of *E. coli* cells to approximate 12 h while the cells were completely killed at 0.125% (*v*/*v*) within 2 h, as shown by transmission electron microscopy [33]. Destruction of the *E. coli* outer and inner membrane might be due to the penetration of the *Litsea cubeba* oil with the observation of many holes and gaps on the damaged cells, which led to killing them eventually. Therefore, a broad application of the *Litsea cubeba* oil in the antimicrobial industry would be possible due to its antimicrobial properties. The time-kill assay of *Foeniculum vulgare* (Fennel) oil against *Shigella dysenteriae* revealed destruction of the membrane integrity [34]. Similarly, it was reported that the leaf essential oil of *Forsythia koreana* acted on the cytoplasmic membrane against food-borne and other pathogenic bacteria, resulting in loss of membrane integrity and increased permeability [35] (Table 2).

Factors determining the activity of essential oils are composition, functional groups present in active components, and their synergistic interactions [41]. The antimicrobial mechanism of action varies with the type of EO or the strain of the microorganism used. It is well known that in comparison to Gram-negative bacteria, Gram-positive bacteria are more susceptible to EOs [42,43]. This can be attributed to the fact that Gram-negative bacteria have an outer membrane which is rigid, rich in lipopolysaccharide (LPS) and more complex, thereby limiting the diffusion of hydrophobic compounds through it, while this extra complex membrane is absent in Gram-positive bacteria which instead are surrounded by a thick peptidoglycan wall not dense enough to resist small antimicrobial molecules, facilitating the access to the cell membrane [24,44]. Moreover, Gram-positive bacteria may ease the infiltration of hydrophobic compounds of EOs due to the lipophilic ends of lipoteichoic acid present in cell membrane [45].

It has been shown in several reports that the bioactive components present in EOs might attach to the surface of the cell, and thereafter penetrate to the phospholipid bilayer of the cell membrane. The structural integrity of cell membrane is disturbed by their accumulation, which can detrimentally influence the cell metabolism causing cell death [46,47]. *E. coli* treated with black pepper essential oil (BPEO) became deformed, pitted, shriveled, because BPEO led to the leakage, disorder and death by breaking cell membrane [36]. Zhang et al. determined the mechanism behind the antibacterial activity of cinnamon EO against *E. coli* and *S. aureus* and reported that the bacterial cell membrane was destroyed after addition of cinnamon EO at the MIC level, whereas addition of cinnamon EO at the MBC levels resulted in the killing of the bacterial cell [37]. In addition to this, cinnamon EO led to increase in the electric conductivity of samples at the first few hours due to leakage of small electrolytes rapidly, concentration of proteins and nucleic acids in cell suspension and 3–5 fold decreased bacterial metabolic activity as reflected by the results of membrane potential. EO from *Dipterocarpus gracilis* inhibited the growth of *Bacillus cereus* and *Proteus mirabilis* by acting on the cytoplasmic membrane as one of its targets. These activities could be exploited for food preservation in the food industry [48]. Further, it has been reported that action of EOs on the integrity of cell membrane changes the membrane permeability which leads to loss of vital intracellular contents like proteins, reducing sugars, ATP and DNA, while inhibiting the energy (ATP) generation and related enzymes leading to the destruction of cell and leakage of electrolytes [49,50]. Antimicrobial activity of EOs is therefore attributed to a cascade of reactions involving the entire bacterial cell [51]. As reported in a study, essential oil from mustard presented 10 times more bactericidal/bacteriostatic effect than cinnamon essential oil [52].

Ahmad et al. [38] showed that antifungal activity of *Coriaria nepalensis* essential oil (CNEO) against *Candida* isolates is due to the inhibition in the biosynthesis of ergosterol and disruption in the integrity of membrane. Similarly, another study described the utility in designing new formulations for candidosis treatment because of the antifungal activity of coriander essential oil on *Candida* spp., in which it was reported that the fungicidal effect of coriander essential oil is a result of damage in the membrane of cytoplasm and subsequent leakage of intracellular components such as DNA [53]. Likewise, disruption of the fungal cell endomembrane system including the plasma membrane and mitochondria, i.e., the inhibition of ergosterol synthesis, malate dehydrogenase, mitochondrial ATPase, and succinate dehydrogenase activities was related to the antifungal activity of natural essential oil (EO) derived from turmeric (*Curcuma longa* L.) against *Aspergillus flavus* [39]. 

## 4. Components of Essential Oils with Antimicrobial Activity

The major constituents of EOs can constitute up to 85%, whereas other components are present in trace amounts [54]. α-phellandrene (36%) and limonene (31%) in *Anethum graveolens* leaf oil, d-limonene (over 80%) in citrus peel oils, α-phellandrene (36%) and limonene (31%) in *Anethum graveolens* leaf oil, carvacrol (30%) and thymol (27%) in *Origanum compactum* oil, α/β-thujone (57%) and camphor (24%) in *Artemisia herba-alba* oil, carvone (58%) and d-limonene (37%) in *Anethum graveolens* seed oil, and menthol (59%) and menthone (19%) in *Mentha piperita* oil are among the constituents present at relatively higher concentrations in essential oils [55]. Generally, the biological properties of the essential oils are determined by their major components including two groups of distinct bio-synthetical origin [56,57]. Terpenes and terpenoids comprise the main groups whereas aromatic and aliphatic constituents comprise the other group, all characterized by low molecular weight.

### 4.1. Terpenes and Terpenoids

**Figure 1:**
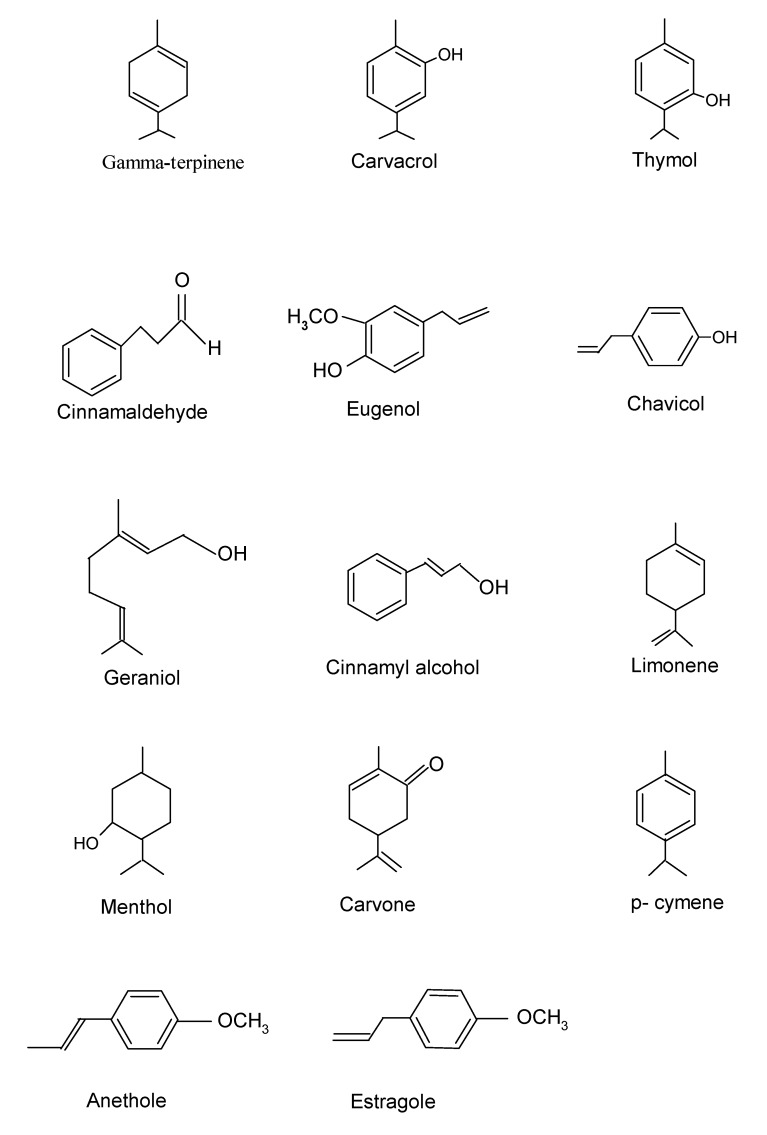
Some representative bioactive compounds present in essential oils.

Several isoprene units (C_5_H_8_) upon combination result in the production of hydrocarbons called terpenes. Occurring in the cytoplasm of plant cells, biosynthesis of terpenes proceeds via the mevalonic acid pathway starting from acetyl-CoA. Having a backbone of hydrocarbons, cyclases can rearrange terpenes into cyclic structures, thus forming monocyclic or bicyclic structures [58]. Terpene biosynthesis consists of synthesis of the isopentenyl diphosphate (IPP) precursor, IPPs being added repetitively to form the prenyldiphosphate precursor of the various classes of terpenes, terpene-specific synthetase modification of the allylic prenyldiphosphate to form the terpene skeleton, and finally, secondary enzymatic modification (redox reaction) of the skeleton to attribute functional properties to the different terpenes [23]. Monoterpenes (C_10_H_16_) and sesquiterpene (C_15_H_24_) are the main terpenes, but longer chains such as diterpenes (C_20_H_32_), triterpenes (C_30_H_40_), etc., also exist. *p*-cymene, limonene, menthol, eugenol, anethole, estragole, geraniol, thymol, γ-terpinene, and cinnamyl alcohol are among the examples of some constituents of essential oils with antimicrobial activity (Figure 1). Angelica, bergamot, lemongrass, mandarin, mint, caraway, celery, citronella, coriander, eucalyptus, geranium, petitgrain, pine, juniper, lavandin, lavander, lemon, orange, peppermint, rosemary, sage, and thyme are among the representatives of plants with some of these compounds [23]. Oxygenated monoterpene (β-fenchol) and oxygenated sesquiterpene (α-eudesmol) were identified as the two main bioactive constituents in the essential oil obtained from fresh leaves of *Eucalyptus teretecornis* with a minimum inhibitory amount (MIA) of 28 μg and 10 μg against *Alternaria alternata* [59]. Similarly, another study reported β-fenchol and linalool as the two antimicrobial components in essential oil obtained from the fresh leaves of *Zanthoxylum alatum* [60]. 

Biochemical modifications of terpenes via enzymes that add oxygen molecules and move or remove methyl groups result in the formation of terpenoids [58]. Terpenoids can be sub-divided into alcohols, phenols, esters, aldehydes, ethers, ketones, and epoxides. Thymol, carvacrol, linalool, linalyl acetate, citronellal, piperitone, menthol, and geraniol are the examples of terpenoids. In one study, *α*-cedrol was reported as the bioactive constituent of the essential oil from fresh leaves of *Thuja orientalis* with a minimum inhibitory amount (MIA) of 30.5 μg against *A. alternate* [61].

Monoterpenoid phenols present in the essential oil of *Origanum vulgare*, thyme, pepperwort and wild bergamot are carvacrol or cymophenol. Diarrheal toxin production by *Bacillus cereus* and growth of vegetative bacteria were inhibited by carvacrol. The precursor of carvacrol is *p*-cymene which is a monoterpene with a benzene ring without any functional groups on its side chains. When used alone, *p*-cymene is not an efficient antimicrobial compound [62,63], but the activity of compounds like carvacrol is potentiated by *p*-cymene [64] and polymyxin B nona peptide [65]. It has been shown that *p*-cymene is hydrophobic in nature and causes swelling of the cytoplasmic membrane to a greater extent [66]. Also, *p*-Cymene had an effect on the synthesis of protein in *E. coli* cells. 

It is expected that the antimicrobial action of phenolic compounds such as thymol and carvacrol is attributed to structural and functional damages in the cytoplasmic membrane [67]. The primary mode of antibacterial action of thymol is not completely understood, but is believed to involve disruption of outer and inner membrane and interaction with membrane proteins and intracellular targets. Thymol (or 2-isopropyl-5-methylphenol), a natural monoterpene phenol derivative of cymene, is isomeric with carvacrol present in thyme essential oil and is extracted from *Thymus vulgaris* (common thyme) and various other plants [68]. In a study by Di Pasqua et al. interaction of thymol with membrane proteins was further supported by exposing *Salmonella enterica* to sub-lethal concentrations of thymol, and accumulation of outer membrane proteins in misfolded pattern and upregulation of genes involved in synthesis of outer membrane proteins was also observed [69]. The citrate metabolic pathway was also impaired by thymol and many enzymes involved directly or indirectly in ATP synthesis. Intracellular action of thymol indicates that it affects important energy-generating processes, which lower the ability of a cell to recover after exposure to thymol. Studies pertaining to investigation of the mode of action of thymol against yeast and fungi point towards the interaction of thymol with the cell envelope and intracellular targets. It has been shown that thymol disrupted vesicles and cell membranes, and impaired biosynthesis of ergosterol in *Candida* strains, which consequently affected the integrity of cell membrane because membrane fluidity and asymmetry is regulated by ergosterol similarly to cholesterol in animal cells [70]. Rao et al. proposed that specific signaling pathways are activated by thymol in yeast, rather than causing non-specific lesion of membranes. This was based on the observation that cytosolic Ca^2+^ bursts caused by thymol and transcription responses similar to those in Ca^2+^ stress and nutrient starvation are activated [71]. Moreover, an increase in the permeability of *P. aeruginosa* and *S. aureus* cells was observed in ethidium bromide (fluorescence nuclear stain), dissipated pH gradients irrespective of glucose availability, and leakage of inorganic ions. These results were in accordance with a study that utilized a mixture of thymol and carvacrol [40].

A major constituent of oregano is carvacrol (a phenolic monoterpenoid). Carvacrol is one of the most extensively studied essential oil constituents together with its closely related isomer thymol. EOs rich in carvacrol have been reported to possess remarkable antimicrobial activity [72,73]. Although the outer membrane is affected by carvacrol, the cytoplasmic membrane is thought to be its site of action, causing passive transport of ions across the membrane. As an adaptation mechanism to maintain optimal membrane function and structure, it has been proposed that cells exposed to carvacrol change the fatty acid composition of the membrane because of the effect of carvacrol on fluidity [74,75]. It has been demonstrated that carvacrol affects the outer membrane of Gram-negative bacteria [76]. 

According to Friedman et al. based on the time they take to produce significant action, essential oils can be divided into the following two types: compounds that act slowly and compounds with fast action. Examples of some antimicrobials considered as fast acting compounds are carvacrol, cinnamaldehyde, and geraniol, since they inactivate organisms like *E. coli* and *Salmonella* in a short time of five minutes. It was reported that a time duration of 30–60 min was required to show efficient antimicrobial activity for the compounds acting slowly [77]. Carvacrols’ mechanism of antifungal activity is similar to thymol, showing H^+^ homeostasis and disruption of Ca^2+^, up- and down-regulation of gene transcription similar to that found in Ca^2+^ stress and nutrient starvation [71], disruption of membrane integrity, and impairment of biosynthesis of ergosterol in *Candida* strains [70]. Silva-Angulo et al. showed that citral exhibited antilisterial activity against *L. innocua* and *L. monocytogenes* and can be applied in active packaging to control possible recontamination of foods or in combination with other preservation technologies [78]. Similarly, Klein et al., determined the antimicrobial activity of six essential oil components against the potential food spoilage bacteria *Aeromonas hydrophila*, *Escherichia coli*, *Brochothrix thermosphacta*, and *Pseudomonas fragi* for single use and in combination with each other [79]. They further showed that, for single use, the most effective oil components were thymol (bacteriostatic effect starting from 40 ppm, bactericidal effect with 100 ppm) and carvacrol (50 ppm/100 ppm), followed by linalool (180 ppm/720 ppm), α-pinene (400 ppm/no bactericidal effect), 1,8-cineol (1400 ppm/2800 ppm), and α-terpineol (600 ppm/no bactericidal effect).

### 4.2. Phenylpropenes

In plants, synthesis of phenylpropenes occurs from the amino acid precursor phenylalanine, constituting a subfamily among the various groups of organic compounds called phenylpropanoids. A relatively small proportion of essential oils is composed of phenylpropenes, and the phenylpropenes that have been most thoroughly studied are safrole, eugenol, isoeugenol, vanillin, and cinnamaldehyde. Eugenol, which is a clear to pale yellow oily liquid is extracted from clove oil, nutmeg, cinnamon, basil, and bay leaves. A study reported eugenol as the antifungal bioactive molecule from *Cinnamomum tamala*, with a minimum inhibitory amount of 9.5 and 8.2 µg against *Alternaria alternata and Curvularia lunata*, respectively [80]. Eugenol has also been shown to cause deterioration of the cell wall, lysis of cells, and prevention of enzyme action in *Enterobacter aerogenes* [81].

The antimicrobial activity of phenylpropenes is dependent on the selected microbial strains, the kind and number of substituents on the aromatic ring, and experimental parameters such as temperature and medium chosen for growth, etc. [82]. Cinnamaldehyde is a flavor- and odor-giving organic compound. Being a pale yellow viscous liquid, it occurs naturally in the bark of cinnamon trees and other species of the genus *Cinnamomum.* It is found as growth inhibitor of *Escherichia coli* and *Salmonella typhimurium* but does not disintegrate the outer membrane or deplete the intracellular ATP pool [81].

## 5. Nano-Encapsulation of Essential Oils for Enhancing Their Antibacterial Effect

A process resulting in the formation of small capsules with many useful properties by surrounding droplets of the bioactive in nature with a coating, or embedding them in a homogeneous or heterogeneous matrix is called encapsulation [83]. Oil encapsulation may retard or even prevent thermo-oxidation reactions, leading to a widening of the intended range of enrichment purposes for food commodities [84]. Bioactive oils are commonly used for their pharmaceutical, cosmetic and nutritional properties. Generally, EOs are volatile substances sensitive to oxygen, light, moisture, and heat. These reported special characteristics could diminish their applicability in cosmetics, food and pharmaceutical industries. Thus, encapsulation is one of the most efficient methods for the formulation of bioactive oils and various studies have been developed in this aspect. The encapsulation system is selected in line with the intended usage of the final formulation, which can vary depending on the size, shape, or nature of selling components. The growing interest in the use of essential oils as natural antimicrobials and preservatives in the food industry has been driven in the last years by the growing consumer demand for natural products with improved microbial safety, and fresh-like organoleptic properties. Nano-emulsions efficiently contribute to support the use of EOs in foods by increasing their dispersibility in the food areas where microorganisms grow and proliferate, by reducing the impact on the quality attributes of the product, as well as by enhancing their antimicrobial activity [85].

Beyki et al. [86] showed that MIC values of free as well as chitosan–cinnamic (CS–Ci) acid nanogel-encapsulated *Mentha piperita* essential oils against *A. flavus* under sealed condition were 2100 and 500 ppm, respectively. Contrary to this, when tested under non-sealed conditions, the encapsulated oils performed better (800 ppm), while within the concentration range tested (up to 3000 ppm) the free oils failed to cause complete inhibition. As a carrier for essential oils in order to enhance their antimicrobial properties, these findings revealed the promising role of CS–Ci nanogel. A higher in vitro bactericidal action of nano-emulsions loaded with essential oils of lemongrass, clove, thyme or palmarosa against *Escherichia coli* has been reported as these nano-emulsions achieved log-reductions of 4.1, 3.6, 2.8 or 3.9, respectively, after a contact time of 30 min. In the case of nano-emulsions containing lemongrass or clove essential oils, faster and enhanced inactivation kinetics were also observed compared to their respective coarse emulsions [87]. Herculano et al. nano-encapsulated *Eucalyptus staigeriana* essential oil (ESO) using cashew gum (CG) as wall material with sizes of nano-emulsions ranging from 27.70 nm to 432.67 nm with negatively charged surfaces. The antimicrobial activity of nanoparticles against *Listeria monocytogenes* (Gram-positive) and *Salmonella Enteritidis* (Gram-negative) was evaluated by determining their minimum bactericidal concentration, The data from MBC showed greater antibacterial activity against Gram-positive bacteria, due to a likely synergistic effect between the ESO and CG. Thus, the data mentioned above suggest that the nanoparticles of ESO have potential to be used as natural food preservatives [88]. Another study revealed superior performance of encapsulated *Zataria multifloria* essential oil (ZEO) by chitosan nanoparticles (CSNPs) under both in vivo and in vitro conditions as compared to unmodified ZEO against *Botrytis cinerea*. The in vivo experiment also showed that at a 1500 ppm concentration, the encapsulated oils significantly decreased both disease severity and incidence of *Botrytis*-inoculated strawberries during 7 days of storage at 4 °C followed by 2–3 days at 20 °C. In another study, the role of CSNPs as a controlled release system for EOs has been suggested [89].

Fatih and Tornuk produced novel water-soluble and thermally stable chitosan nanoparticles loaded with different levels (1%, 1.2%, 1.4% and 1.5%) of summer savory (*Satureja hortensis* L.) essential oil using an ionic gelation method. NPs loaded with essential oils exhibited strong antibacterial activity against *Staphylococcus aureus*, *Listeria monocytogenes* and *Escherichia coli* depending on the concentration of EO encapsulated. It was concluded that the summer savory EO-loaded chitosan NPs were highly adapted to excessive environmental factors such as high temperature and acidic pH, possessing high bioactive properties convenient for future food processing and packaging applications [90]. Also, Shengjiang et al. prepared blended cloves/cinnamon essential oil nano-emulsions using Tween 80 and ethanol as surfactant and co-surfactant, respectively. Even at far lower concentrations, the nano-emulsion showed higher antimicrobial activity against the four tested microorganisms *Escherichia coli*, *Bacillus subtilis*, *Salmonella typhimurium*, and *Staphylococcus aureus*. This shows that blended cloves/cinnamon essential oil nano-emulsions have the potential to be a natural antimicrobial agent in the food industry [91].

Mohammadi et al. [92] studied the performance of *Cinnamomum zeylanicum* essential oil (CEO) when encapsulated by CSNPs under both in vitro and in vivo conditions in comparison with unmodified CEO against *Phytophthora drechsleri*. The in vivo study showed that at a concentration of 1.5 g/L the encapsulated oils of *Cinnamomum zeylanicum* significantly decreased both disease severity as well as incidence of *Phytophthora* in inoculated cucumbers over 7 days of storage at 4 °C followed by 2–3 more days at 20 °C. Furthermore, the shelf life of cucumbers with CEO-CSN coating was extended up to 21 days at 10 ± 1 °C while uncoated fruit were unmarketable in less than 15 days. In addition to this, CEO-CSN coated fruits were firmer, maintained color, water content, had improved microbiological and physicochemical quality and showed lower microbial counts throughout storage. Thus, CEO-CSN coatings can be an effective method to extend cucumber shelf life. Similarly, Guerra-Rosas et al. assessed the antimicrobial activity of nano-emulsions containing oregano, thyme, lemongrass or mandarin essential oils and high methoxyl pectin during a long-term storage period (56 days) against *E. coli* and *Listeria innocua*. Regardless of the EO type, a higher antimicrobial activity was detected against *E. coli* as compared to *L. innocua*. Significant damage in the *E. coli* cells for both the cytoplasm and cytoplasmic membrane led to cell death which was revealed by the images of transmission electron microscopy (TEM). The antimicrobial activity of the nano-emulsions was found to be strongly related to the EO type rather than to their droplet size. The smallest droplet size (11 ± 1 nm) of the lemongrass-pectin nano-emulsion had a higher antimicrobial activity, reaching 5.9 log reductions of the *E. coli* population. Nevertheless, nano-emulsion of the freshly-made oregano, thyme and mandarin EO–pectin led to 2.2, 2.1 or 1.9 *E. coli* log-reductions, respectively. However, a significant decrease in the antimicrobial activity was observed during storage regardless of the EO type, which was related to the loss of volatile compounds over time [93]. Besides EOs, there are numerous reports available on the antimicrobial efficacy of nanoparticles. Prabhu and Poulose reviewed various mechanisms of antimicrobial action of NPs, especially silver NPs [94], which are potential antimicrobial agents [95]. Silver NPs create pits and thus anchor and penetrate the cell wall, causing release of free radicals followed by structural change in the cell membrane leading to a greater influx of the antibacterial agent through cytoplasmic membrane because of increased cell permeability, resulting in cell death. Another mechanism of the silver nanoparticles is the formation of free radicals, by which the cells die. It has been suggested by electron spin resonance spectroscopy studies that silver nanoparticles form free radicals, when in contact with bacteria, and these free radicals have the ability to make the cell membrane porous by damaging it which can ultimately lead to cell death [96]. Moreover, silver nanoparticles attacking DNA bases can also inhibit signal transduction and cell wall formation, and interact with respiratory enzymes, liberating reactive oxygen species which is followed by cell death.

Over the last decade, several research studies have focused on the synergy between EOs and various types of NPs for their superior antimicrobial efficacy. Cinnamaldehyde, a representative of EO, showed a strong synergistic activity with silver NPs against spore-forming *Bacillus cereus* and *Clostridium perfringens*. Bacterial kill curve analysis revealed rapid bactericidal action exerted by this combination of antimicrobial agents, while extensive damage to the cell envelope was evidenced by electron and atomic force microscopy [97].

Release of silver ions by nanoparticles can inactivate many enzymes by interacting with the thiol groups [98,99]. An enhancement in the antimicrobial potential of EOs can be attained by encapsulating with various nanomaterials e.g., solid lipid NPs, liposomes, polymeric NPs and nano-emulsions, where the inside core consists of EO while nanomaterial forms the outer nano-capsule. Essential oils upon nano-encapsulation exhibit physical stability, decreased volatility and protection from environmental interactions (e.g., light, oxygen, moisture, pH), enhanced bioactivity and reduced toxicity [100]. Small nano-emulsion droplets are able to bring the EOs close to the cell membrane surface, improving the accessibility to microbial cells and enabling the membranes of the cells to be disrupted, possibly by altering the integrity of phospholipid bilayer or by interfering with the embedded phospholipid bilayer active transport proteins [101]. In order to modulate drug release i.e., burst release and/or controlled release, this represents a promising approach [102]. The in vitro release study of encapsulated oregano EO with chitosan NPs revealed an initial burst effect followed by slow release of drug (EO) [103,104]. Similarly, efficient biocidal activity against *Stegomyia aegypti* larvae was observed due to a slow and sustained release of EO by chitosan/ cashew gum nano-encapsulation. The effect of EO nano-emulsions on yeast cells has also been addressed in several studies among which *Zygosaccharomyces bailii* and *Saccharomyces cerevisiae* are the most investigated. Yeast cells required longer incubation times with respect to bacterial inactivation, when exposed to carvacrol, cinnamaldehyde, and d-limonene nano-emulsions [105], and exhibited lower minimum inhibitory concentration for encapsulated d-limonene [106]. In another study, an enhancement in the antimicrobial activity of carvacrol loaded in polylactic glycolic acid nano-capsules was reported due to significant transformation in the rheological characteristics of bacterial biofilm that potentially facilitated the activity of carvacrol [107]. EOs are protected from enzymatic degradation by the nano-carriers which transform them into powder and help to achieve the desired therapeutic levels for the required time duration to the target tissues with reduction in number of doses and may also ensure an optimal pharmacokinetic profile [102]. Thus, the combination of various EOs with their inherent antimicrobial activity with other potent antimicrobial agents like NPs may greatly enhance their antimicrobial activity by complementing each other with the involvement of various mechanisms against different types of pathogens. Therefore, combinations of different antimicrobials appear to be the best strategy for controlling multidrug resistant microbes.

## 6. Study of Synergistic Antimicrobial Activity of Essential Oils

There is a need to find alternative strategies to deal with infections resulting from drug-resistant bacteria, due to an increase in antibiotic-resistant bacteria and the lack of new antibiotics being brought onto the market. Development of alternatives to antibiotics and the discovery or development of adjuvants are amongst the potential strategies proposed [27]. Combination of antibiotics with other drugs that are non-antibiotic is one such possibility [108]. Another such possibility is the combination of antibiotics with adjuvants or antimicrobials selected from nature’s reservoir of bioactive compounds [27]. An overview pertaining to synergism between plant metabolites and antibiotics has been provided by Hemaiswarya et al. and according to them promising adjuvants of antibiotics may be represented by phytochemicals [109]. Essential oils (EOs) and their components form part of the group of phytochemicals that is said to have such effects, according to in vitro studies [110].

In a combination, the interaction between antimicrobials can result in three different outcomes i.e., synergistic, additive, or antagonistic. Synergy is obtained by combining two antimicrobial compounds producing antibacterial activity greater than the sum of the antibacterial activity of individual components. An additive effect is produced by combining antimicrobials producing an antimicrobial effect that is equal to the sum of the individual compounds. An antagonistic effect results in a decreased antimicrobial activity of two compounds in combination as compared with their individual antimicrobial activity [27]. For calculating the index of the fractional inhibition concentration, measurements of the MIC are used for the analysis of the combined effect of a blend (FIC index) according to the formulas defined by Davidson and Parish: FICA = MICA + B/MICA, FICB = MICB + A/MICB, FIC index = FICA + FICB. The MIC of compound A in the presence of compound B is represented by the MICA + B value and vice versa for MICB + A. Determination of the MIC for the individual components is required for calculating the FIC value for either substance A or B. Theoretically, an FIC index near one indicates additive interactions, while below one implicates synergistic interactions, and above one indicates antagonistic interactions [111]. However, a more general definition has replaced this aforementioned definition of FIC, according to which the results of the FIC index are interpreted as synergistic if the FIC index < 0.5, additive if 0.5 < FIC index < 4, or antagonistic if the FIC index > 4 [112]. Synergy can be discussed in the following four mechanisms based on results of the latest investigations in classic pharmacological, molecular–biological and clinical works.

Synergistic effects can be produced if the constituents of an extract affect different targets or interact with one another in order to improve the solubility and thereby enhance the bioavailability of one and several substances of an extract. Further, when antibiotics are combined with an agent that antagonizes bacterial resistance mechanisms can lead to synergy [113].

### 6.1. Synergism between Components of Essential Oils

Antimicrobial activity of a given essential oil may depend on one or two of the major constituents only that make up the entire oil. In accordance with the increasing level of evidence, the ratio in which the main active constituents are present may not be the only factor responsible for the inherent activity of essential oils, but the interactions between these and minor constituents in the oils are also important. According to Bassolé et al. the combination of eugenol with linalool or menthol exhibited the highest synergy, suggesting that combination of a monoterpenoid phenol with a monoterpenoid alcohol is effective [114]. When tested in binary or ternary combinations, various synergistic antimicrobial activities have been reported for constituents or fractions of essential oils [115,116]. For example, García-García et al. found carvacrol and thymol to be the most synergistic binary combination against *L. innocua*, and the carvacrol, thymol, and eugenol to be the ternary combination that was most active [115]. 

### 6.2. Synergism between Different Essential Oils

Bag and Chattopadhya showed that the coriander/cumin seed oil combination showed synergistic antibacterial interactions with an FICI ranging from 0.25 to 0.5 [117]. In a study by Hossain et al. eight essential oils (EOs) of plants, namely, eucalyptus, tea tree, basil, oregano, cinnamon, mandarin, peppermint, and thyme were evaluated for their ability to inhibit growth of *Aspergillus niger*, *Penicillium chrysogenum*, *Aspergillus flavus*, and *Aspergillus parasiticus* and it was reported that a combined formulation of oregano with thyme essential oil resulted in a synergistic effect, thus showing an enhancement in the efficiency against *A. flavus*, *A. parasiticus* and *P. chrysogenum*. A synergistic effect was also exhibited by mixtures of peppermint and tea tree essential oil against *A. niger*, and thyme and cinnamon against *A. flavus*. Also, a synergistic effect was observed against *P. chrysogenum* by combining oregano essential oil with essential oils of cinnamon, tea tree, thyme and mint, and an individual mixture of mint with thyme [118]. Similarly, a synergistic effect against *A. flavus* was produced by combination of oregano and thyme, cinnamon and thyme and oregano and mint essential oils. Also, combination of mint with tea tree oil showed a synergistic effect against *A. niger*. It was revealed that the combination of some particular oils produced synergism as a result of the combined activities of two or more constituents of essential oils. Because pathogens cannot easily acquire resistance to multiple components of two or more essential oils, such an increase in the fungistatic activity would be advantageous in pre- and post-harvest protection [119].

### 6.3. Synergism between Components/Constituents of Essential Oils and Antibiotics

Components of essential oils namely thymol and carvacrol were found to show synergism with penicillin against *E. coli* and *S. typhimurium* [120]. Similarly, cinnamaldehyde, that possesses a prop-2-enal side group to the benzene ring, had synergistic activity with fewer of the antibiotics as compared to the phenols carvacrol and thymol [120,121], which could provide some initial indications on the mechanism of these components of volatile oils. An examination of the synergistic action between eugenol and antibiotics against a number of reference strains of cariogenic and period onto pathogenic bacteria has also been carried out. It was reported that eugenol exhibited synergism with ampicillin against *S. criceti* and *Streptococcus gordonii* and with gentamicin against *Streptococcus sanguinis* and *Porphyromonas gingivalis* [122]. However, carvacrol was found to be synergistic in combination with both ampicillin and nitrofurantoin against *Klebsiella oxytoca* that was isolated from animal feed whilst thymol was indifferent [121].

### 6.4. Synergism between Essential Oils and Antibiotics

In a study conducted by Rosato et al. it was reported that oregano oil in combination with gentamicin exhibited synergism against *B. cereus*, *B. subtilis* and one strain of *S. aureus* [123]. *Zataria multiflora* (Shiraz oregano) essential oil exhibited synergistic activity with vancomycin against methicillin-sensitive *Staphylococcus aureus* (MSSA) and 12 clinical isolates of MRSA, although the FIC data for individual strains were not stated [124]. In another study, Australian tea tree (*Melaleuca alternifolia*) volatile oil combinations with aminoglycoside antibiotics were investigated. *E. coli*, *Yersinia enterocolitica*, *Serratia. marcescens* and one strain of *S. aureus* were among the bacterial species for which synergism was found with gentamicin [125]. The FIC index was found to be at borderline between additivity and synergism against *Acinetobacter baumannii*, *B. subtilis* and another strain of S. aureus [123]. Also, tea tree oil along with tobramycin had synergism against *E. coli* and *S. aureus* [32]. It was found that aminoglycosides inhibit protein synthesis and tea tree oil damages the bacterial cytoplasmic membrane; this was possibly an example of multi-target synergy. Ampicillin and gentamicin along with clove oil has been tested for synergism against a number of periodontic pathogens. FIC indices of less than 0.5 were found for ampicillin against *Streptococcus mutans*, *S. sobrinus* and *Streptococcus gordonii* and for gentamicin against *Streptococcus sanguinis*, *S. criteci* and *Porphyromonas gingivalis* [120]).

In an in vitro study conducted by Duarte et al. the combination of coriander essential oil with gentamicin, chloramphenicol, ciprofloxacin, and tetracycline against *Acinetobacter baumannii* showed effectiveness, which was therefore an indicator of a possible synergistic interaction against two reference strains of *Acinetobacter baumannii* (LMG 1025 and LMG 1041) with an FIC index of 0.047 and 0.37, respectively. This study indicated that this in vitro interaction could improve the antimicrobial effectiveness of tetracycline, ciprofloxacin and gentamicin and may contribute to re-sensitize *Acinetobacter baumannii* for the action of chloramphenicol [125].

In most of the cases, examination of combination of *Eucalyptus camaldulensis* essential oils with conventional antibiotics (gentamycin, ciprofloxacin, and polymyxin B) showed synergistic antibacterial effect even in some re-sensitized multidrug-resistant (MDR) *A. baumannii strains*. Time-kill curves confirmed the synergistic interaction of *E. camaldulensis* essential oil and polymyxin B combination resulting in a reduced bacterial count under very fast detection limit, i.e., after 6 h of incubation [126].

Boonyanugomol et al. determined the antibacterial and synergistic activities of the *Zingiber cassumunar* essential oil against the extensively drug-resistant (XDR) *Acinetobacter baumannii* strains. A synergistic effect was produced by combining the essential oil with antibiotics, e.g., aminoglycosides, tetracyclines, fluoroquinolones, and folate pathway inhibitors which may provide the basis for the development of a new therapy against drug-resistant bacteria [127].

## 7. Conclusions

Evidence of increasing of multidrug resistance in pathogens at an alarming pace is seen in the high rates of morbidity and mortality. It is one of the great challenges faced by clinicians and researchers. The inefficacy of existing medical treatments has necessitated the search for novel and efficient drugs to tackle this problem. Essential oils possess important volatile compounds with diverse bioactivities including antimicrobial potential. EOs have been used in drugs, food, and cosmetics due to these properties. However, there are certain limitations, such as strong organoleptic flavor, low water solubility and low stability. The antimicrobial properties of EOs are mostly related to the individual susceptibility of bacteria. The promising antimicrobial activity of EOs has encouraged researchers to use them along with nanomaterials, essential oils of other plants, essential oil components, and antibiotics as potential antimicrobial agents. Encapsulation simultaneously increases the antimicrobial potency of essential oils by controlled/sustained release and facilitating close interaction with the microorganisms. There is a lack of detailed knowledge about the mechanism of the individual essential oil components which thus attributes to our superficial understanding of governing synergy and antagonism. Therefore, research in the future should thus explore the mechanism of individual essential oil components, along with an initiation in systematically investigating the synergy mechanisms among different components. Therefore, new strategies for nano-encapsulation and synergistic studies can provide an interesting platform in the near future for this area of research.

## Figures and Tables

**Table 1:** Minimum inhibitory concentration (MIC) values of some essential oils against different bacteria.

Plant from Which Essential Oil is Derived	Micro-Organisms	MIC Values	References
*Cymbopogan citratus*	*Escherichia coli*	0.6 µL/mL	[11]
	*Salmonella typhimurium*	2.5 µL/mL	
	*Staphylococcus aureus*	0.6 µL/mL	
*Satureja Montana*	*Pseudomonas aeruginosa*, *Streptococcus pyogenes*, *Streptococcus mutans*, *Streptococcus sanguis*, *Streptococcus salivarius*, *Enterecoccus feacalis Lactobacillus acidophilus*	23.33 ± 5.77 µg/mL 116.67 ± 15.28 µg/mL 60.00 ± 0.00 µg/mL 23.33 ± 7.64 µg/mL 23.33 ± 5.77 µg/mL 53.33 ± 5.77 µg/mL 125.00 ± 8.66 µg/mL	[18]
*Origanum vulgarae*	*Escherichia coli*	1600–1800 ppm	[19]
	*Staphylococcus aureus*	800–900 ppm	
*Lavandula officinalis*	*Escherichia coli*	2000 ppm	[19]
	*Staphylococcus aureus*	1000–1200 ppm	
*Cinnamomum zeylanicum*	*Acinetobacter*	8 mg/mL	[20]
	*Klebsiella pneumoniae*	2 mg/mL	
	*Protreus vulgaris*	8 mg/mL	
	*Enterococcus fecalis*	4 mg/mL	
	*Staphylococcus aureus*	0.5 mg/mL	
	*Staphylococcus epidermidis*	1 mg/mL	
*Psiadia arguta*	*Acinetobacter*	16 mg/mL	[20]
	*Enterococcus fecalis*	8 mg/mL	
	*Staphylococcus aureus*	0.5 mg/mL	
	*Staphylococcus epidermidis*	0.25 mg/mL	
*Piper betle*	*Acinetobacter*	8 mg/mL	[20]
	*Klebsiella pneumonia*	4 mg/mL	
	*Proteus vulgaris*	4 mg/mL	
	*Enterecoccus fecalis*	4 mg/mL	
	*Staphylococcus aureus*	0.5 mg/mL	
	*Staphylococcus epidermidis*	0.5 mg/mL	
*Pimenta dioica*	*Acinetobacter*	8 mg/mL	[20]
	*Klebsiella pneumoniae*	4 mg/mL	
	*Enterecoccus fecalis*	2.5 mg/mL	
	*Staphylococcus aureus*	1 mg/mL	
	*Staphylococcus epidermidis*	1 mg/mL	
*Psiadia terebinthina*	*Acinetobacter*	16 mg/mL	[20]
	*Klebsiella pneumoniae*	4 mg/mL	
	*Proteus vulgaris*	8 mg/mL	
	*Enterecoccus fecalis*	8 mg/mL	
	*Staphylococcus aureus*	0.5 mg/mL	
	*Staphylococcus epidermidis*	0.25 mg/mL	
*Ocimum basilicum*	*Clostridium perfringens*	5.0 mg/mL	[21]
*Rosmarinus officinalis*		10 mg/mL	
*Origanum majorana*		5.0 mg/mL	
*Mentha piperita*		10 mg/mL	
*Thymus vulgaris*		1.25 mg/mL	
*Pimpinella anisum*		10 mg/mL	
*Epilobium parviflorum*	*Enterecoccus fecalis*, *Staphylococcus aureus*, *Escherichia coli and Pseudomonas aeruginosa*	10–40 µg/mL	[22]
*Salvia desoleana*	*Staphylococcus aureus*	2 or >2 mg/mL	[23]
*Salvia sclarea*	*Staphylococcus epidermidis*	1.5–2 mg/mL	[23]
*Allium sativum*	*Escherichia coli*	15–1500 µg/mL	[24]
*Cuminum cyminum*	*Bacillus cereus*	0.05 µL/mL	[24]
	*Bacillus subtilis*	1000 µg/mL	
*Ocimum gratissimum*	*Escherichia coli*	6 µg/mL	[24]
	*Pseudomonas aeruginosa*	≥24 µg/mL	
	*Staphylococcus aureus*	0.75 µg/mL	

**Table 2:** Mechanism of action of certain essential oils against different micro-organisms.

Plant from Which Essential Oil is Derived	Micro-Organism Targeted	Mechanism of Action	Reference
*Allium sativum*	*Escherichia coli*	Induced leakage	[24]
*Litsea cubeba*	*Escherichia coli*	Destruction of outer and inner membrane	[33]
*Foeniculum vulgare*	*Shigella dysenteriae*	Loss of membrane integrity	[34]
*Forsythia koreana*	Foodborne and other pathogenic bacteria	Loss of membrane integrity and increased permeability	[35]
*Piper nigrum*	*Escherichia coli*	Cell becomes pitted, shriveled and leakage of intercellular material.	[36]
*Cuminum cyminum*	*Bacillus cereus* *Bacillus subtilis*	Changes in cytoplasm	[24]
Cinnamon	*Escherichia coli* *Staphylococcus aureus*	Disruption of cell membrane	[37]
*Dipterocarpus gracilis*	*Bacillus cereus* *Proteus mirabilis*	Disruption of cell membrane.	[37]
*Ocimum gratissimum*	*Escherichia coli* *Pseudomonas aeruginosa* *Staphylococcus aureus*	Permeabilized membrane	[24]
*Coriaria nepalensis*	*Candida* isolates	Inhibition of ergosterol biosynthesis and disruption of membrane integrity	[38]
*Curcuma longa*	*Aspergillus flavus*	Inhibition of ergosterol biosynthesis	[39]
*Origanum vulgare*	*Escherichia coli* *Staphylococcus aureus* *Pseudomonas aeruginosa*	Permeabilized membrane	[40]
*Mentha longifolia*	*Escherichia coli* *Micrococcus luteus* *Salmonella typhimurium*	Cell wall damage	[24]
